# High prevalence and emerging cephalosporin resistance of penicillinase-producing *Neisseria gonorrhoeae* in Meizhou, China (2022–2024)

**DOI:** 10.1186/s12879-025-12030-x

**Published:** 2025-11-14

**Authors:** Qian Li, Xueqin Chen, Yuhong Huang, Jinyan Wu, Zhaoye Huang, Xinlu Wang, Xuemin Guo

**Affiliations:** 1 Clinical Laboratory Center, Meizhou People’s Hospital, Meizhou, Guangdong China; 2 Medical College of Jiaying University, Meizhou, Guangdong China; 3 State Key Laboratory of Biomacromolecules, Institute of Biophysics, Chinese Academy of Sciences, Beijing, China; 4 Guangdong Engineering Technological Research Center of Clinical Molecular Diagnosis and Antibody Drugs, Meizhou, Guangdong China; 5 Guangdong Provincial Clinical Research Center for Laboratory Medicine, Guangzhou, Guangdong China

**Keywords:** Penicillinase-producing *Neisseria gonorrhoeae*, Plasmid typing, *bla*_TEM−135_, NG-MAST

## Abstract

**Background:**

The global spread of penicillinase-producing *Neisseria gonorrhoeae* (PPNG) has rendered penicillin-based regimens largely ineffective, and emerging cephalosporin-resistance patterns continue to challenge therapeutic strategies. However, epidemiological surveillance data from resource-limited regions of China remain scarce. This study aimed to determine the prevalence, molecular epidemiology and cephalosporin susceptibility of PPNG isolates in Meizhou, China.

**Methods:**

*Neisseria gonorrhoeae* isolates were collected from Meizhou People’s Hospital between 2022 and 2024. Antimicrobial susceptibility to penicillin, ceftriaxone, and cefixime was determined using the broth microdilution method, with PPNG identified by the nitrocefin test. Plasmid types were detected by multiplex PCR, while *bla*_TEM_ genes were analyzed using Mismatch Amplification Mutation Analysis (MAMA). Full-length sequencing of the *penA* gene was performed for ceftriaxone/cefixime-resistant isolates. All PPNG strains underwent molecular typing via *Neisseria gonorrhoeae* multiantigen sequence typing (NG-MAST) with phylogenetic tree construction, while *bla*_TEM−135_-carring strains were further analyzed through *porB*-based phylogenetic reconstruction.

**Results:**

A total of 101 *Neisseria gonorrhoeae* isolates were collected. Among these, 78.2% (79/101) were PPNG, carrying African-type (92.4%, 73/79) or Asian-type (7.6%, 6/79) plasmids; within the PPNG group, 94.9% (75/79) harbored *bla*_TEM−1_ and 5.1% (4/79) *bla*_TEM−135_. African-type *bla*_TEM−1_-positive PPNG isolates showed cephalosporin-resistance rates similar to those of non-PPNG isolates for both ceftriaxone (12.3% vs. 9.1%; *p* = 1.00) and cefixime (20.5% vs. 13.6%; *p* = 0.76), with no statistically significant difference. Overall, 17.7% of the PPNG strains exhibited reduced cephalosporin susceptibility (MIC > 0.125 mg/L) and harbored either mosaic or non-mosaic PBP2 patterns. NG-MAST identified 43 sequence types, with ST17748 being the predominant ST. Phylogenetic analysis revealed a dispersed distribution of Asian-type *bla*_TEM−135_ strains, and *bla*_TEM−135_-positive isolates clustered closely with epidemic strains from Japan, Thailand, and Chinese cities.

**Conclusion:**

The high prevalence of PPNG and emerging cephalosporin resistance, including *bla*_TEM−135_ variants with potential ESBL activity, highlights the need to strengthen regional surveillance and develop appropriate control measures for resistant gonorrhea in this area.

**Clinical trial number:**

Not applicable.

## Introduction

Gonorrhea is one of the most prevalent sexually transmitted infections and poses a significant public health challenge. The World Health Organization (WHO) estimated approximately 82.4 million cases of *Neisseria gonorrhoeae* infection worldwide in 2020 [1]. In China, a total of 96,313 cases of gonorrhea were reported, ranking it as the fourth most commonly reported notifiable infectious disease in the country [2]. Untreated *Neisseria gonorrhoeae* infection can lead to severe complications, including pelvic inflammatory disease, ectopic pregnancy, infertility, and an increased susceptibility to HIV infection [3]. Without an available vaccine, the management of gonorrhea depends on effective antimicrobial therapies.

Penicillin was the first-line treatment for gonorrhea until the emergence of penicillinase-producing *Neisseria gonorrhoeae* (PPNG), which spread globally following its initial detection [4]. This strain expresses a class A β-lactamase encoded by the *bla*_TEM−1_ gene, which hydrolyzes the cyclic amide bond of the β-lactam ring in penicillin [5]. The genetic determinant for this enzyme is carried on multiple related plasmids. To date, eight plasmid types have been characterized and classified based on their epidemiological origins; these include Asian, African, and Toronto/Rio plasmids, along with other rare variations such as Nimes, New Zealand, Johannesburg, and Australian plasmids [6]. These plasmids carrying the β-lactamase gene evolve via insertion or deletion of DNA fragments [7]. This type of PPNG strain carries plasmids that mediate high levels of penicillin resistance and play an important role in epidemiology [8–13]. Coupled with the emergence of chromosomally mediated resistance to penicillin, this development led to the abandonment of this once highly effective antimicrobial agent [5].

Currently, the preferred drugs for treating gonorrhea are third-generation cephalosporins from the β-lactam class, particularly ceftriaxone and cefixime, which can be used alone or in combination with the macrolide antibiotic azithromycin [4]. However, treatment failures due to *Neisseria gonorrhoeae* exhibiting reduced susceptibility or resistance to ceftriaxone have been reported in many countries worldwide [14, 15]. Chromosomal mutations represent the primary factor contributing to cephalosporin resistance. Equally concerning is the potential for plasmid-mediated mutations in the penicillinase-encoding *bla*_TEM_ β-lactamase gene [5]. In 2004, a PPNG isolate carrying the *bla*_TEM−135_ gene was identified in Japan. TEM-135 differs from TEM-1 by only one single nucleotide polymorphism (SNP). The substitution of thymine with cytosine at position 539 results in a single amino acid change at position 182, replacing methionine with threonine [16]. This gene sequence is identical to that found in Salmonella enterica [17]. A particular concern is that TEM-135 requires only a single amino acid substitution to become an extended-spectrum β-lactamase (ESBL) [10–12, 16], which would degrade nearly all extended-spectrum cephalosporins and may abandon the use of third-generation cephalosporins. PPNG isolates carrying the *bla*_TEM−135_ gene have been identified worldwide. Although ESBL has not yet been detected in *Neisseria gonorrhoeae*, research suggests TEM-135 may serve as a potential precursor that could evolve into more potent drug resistance [8, 10–13]. Therefore, enhanced surveillance of TEM-135 holds critical importance for *Neisseria gonorrhoeae* prevention and control.

Meizhou, located at the junction of three mountainous provinces in northeastern Guangdong Province and adjacent to the highly developed Pearl River Delta region, experiences a significant flow of population between urban and rural areas. Combined with evolving social norms and emerging public health challenges, this has made the control of gonorrhea in this region particularly challenging. In 2022, Guangdong Province reported a total of 22,171 cases of *Neisseria gonorrhoeae* infection [2], but no specific data were available for Meizhou, and related research remains scarce. As a sentinel surveillance site of the Guangdong Gonococcal Antimicrobial Surveillance Program (GD-GASP) and a core institution for regional sexually transmitted disease (STD) prevention and control, Meizhou People’s Hospital is responsible for the majority of STD screening in the region. This study systematically analyzed *Neisseria gonorrhoeae* clinical isolates collected from the hospital between 2022 and 2024, aiming to provide crucial reference data for understanding the epidemic trend of *Neisseria gonorrhoeae* in Meizhou.

## Methods

### *Neisseria gonorrhoeae* isolates

This study retrospectively analyzed *Neisseria gonorrhoeae* isolates obtained from Meizhou People’s Hospital (a Grade-A tertiary general hospital in Guangdong Province) between January 2022 and December 2024. All isolates were derived from clinical specimens (including urethral and cervical secretions) collected from patients with suspected genital tract infections. The clinical specimens were cultured on Thayer-Martin agar and incubated in a 5% CO₂ incubator at 35 °C for 24–48 h. The isolates were identified by matrix-assisted laser desorption/ionization time-of-flight mass spectrometry (MALDI-TOF MS, Bruker Daltonics, Germany). The nitrocefin disk test (Pangtong Medical, Chongqing, China) was used to detect β-lactamase production. All strains were preserved in freeze-dried skimmed milk and stored at − 70 ℃ until further use. The WHO strains G, J, P, and ATCC49226 supplied by the Guangdong Provincial Center for Skin Disease and Sexually Transmitted Infection Control and Prevention served as quality controls.

### Antibiotic susceptibility testing

The minimum inhibitory concentration (MIC) of *Neisseria gonorrhoeae* was determined using the broth microdilution method (Jinbiao Medical, Zhuhai, China). Previous studies have confirmed that this method has a concordance rate of over 93.5% with the agar dilution method for β-lactams (98.4% for ceftriaxone), quinolones, and macrolides, demonstrating equivalent capability in discriminating resistance [18–20]. A pure culture suspension was prepared in 0.45% saline and adjusted to 0.5 McFarland as inoculum. The bacterial suspensions were inoculated into the M-H broth medium, thoroughly mixed, and subsequently added onto drug-sensitive dilution strips. The strips were then incubated at 35 ℃ in a 5% CO_2_ incubator for 24 h. Bacterial growth was subsequently evaluated, and the results were interpreted according to the European Committee on Antimicrobial Susceptibility Testing (EUCAST) criteria [21]. Strains with MICs>1.0 mg/L for penicillin were classified as resistant. Similarly, strains with MICs>0.125 mg/L for ceftriaxone or cefixime were categorized as resistant. The reference strains, the WHO strains G, J, P, and ATCC49226, were used as quality control.

### Plasmid typing and genotyping of PPNG

For genomic DNA extraction, the appropriate colonies were suspended in PBS buffer (pH 7.0, Solarbio, Beijing, China), boiled for 10 min, and then placed in an ice bath for 5 min. The supernatant was obtained after centrifugation at 12,000 r/min for 10 min and subsequently stored at − 20 ℃ for later use.

The plasmids encoding β-lactamase were characterized by multiplex PCR using specific forward and reverse primers [22]. Subsequently, the specifically sized PCR products were identified by electrophoresis on a 1% agarose gel containing a 1000-bp DNA ladder (Accurate Biology, Changsha, China) in every run; amplicon sizes were read directly from the ladder, allowing unambiguous assignment to Asia-type (958 bp), Africa-type (1,191 bp), or Toronto/Rio-type (650 bp) plasmids. The identification of the *bla*TEM-135 gene was achieved by performing a mismatch amplification mutation assay (MAMA) polymerase chain reaction (PCR) as previously described [23]. Each PCR assay included positive controls comprising DNA extracts from reference strains MZ003 (containing African-type plasmid with *bla*_TEM−1_) and MZ035 (containing Asian-type plasmid with *bla*_TEM−135_). All primer synthesis was performed by Sangon Biotech (Shanghai, China).

### *penA* gene of PPNG

To analyze the molecular mechanisms underlying chromosomally mediated resistance to penicillin and cephalosporins, such as ceftriaxone and cefixime, we performed full-length sequencing of the *penA* gene encoding penicillin-binding protein 2 (PBP2) in PPNG isolates resistant to ceftriaxone and/or cefixime (MIC >0.125 mg/L), as described in reference [24]. The nucleotide and amino acid sequences of PBP2 from PPNG isolates were compared with those of penicillin-susceptible *Neisseria gonorrhoeae* LM306 (GenBank accession no. M32091) and classified into sequence patterns as previously reported [25].

### Molecular typing of PPNG

*Neisseria gonorrhoeae* multiantigen sequence typing (NG-MAST) was carried out by amplifying and sequencing the *porB* and *tbpB* genes according to the protocol of Martin et al. [26]. The amplified products were identified by agarose-gel electrophoresis and then sent to Sangon Biotech for Sanger sequencing. The resulting sequences were queried against the PubMLST Neisseria database (https://pubmlst.org/neisseria/) to obtain allele and sequence type (ST) numbers. For strains with unsuccessful query results, the allele information would be submitted to the PubMLST website to apply for a new sequence type.

### Phylogenetic analysis

To allow for the visualisation of *Neisseria gonorrhoeae* sequence diversity, we first analysed concatenated sequences of NG-MAST trimmed *porB* and *tbpB* sequences. Multiple alignments of all sequences and construction of the neighbour-joining tree based on Kimura’s two-parameter method were performed using the ClustalW tool implemented in MEGA11 [27]. Tree annotation and optimization were carried out by using the online platform chiplot (https://www.chiplot.online/) [28].

Furthermore, to investigate the phylogenetic relationships among PPNG isolates harboring *bla*_TEM−135_, a phylogenetic tree was constructed based on partial *porB* gene sequences from the isolates possessing *bla*_TEM−135_ in this study and compared with those reported in previous studies involving domestic and international isolates. The *porB* sequences of isolates were downloaded from the NG-MAST database according to the STs specified in the references [8, 12, 13, 23, 29]. Phylogenetic analysis was performed as previously described.

### Statistical analyses

The Fisher’s exact test was used to analyze the antibiotic resistance rates of different groups of *Neisseria gonorrhoeae*, while the chi-square test was employed to assess the significance of regional differences in PPNG prevalence. A two-tailed *P*-value < 0.05 was considered statistically significant. All statistical analyses were performed using IBM SPSS Statistics version 22.0.

## Results

### Prevalence of PPNG, plasmid type, and *bla*_TEM_ gene

A total of 101 *Neisseria gonorrhoeae* were isolated, of which 79 strains (78.2%, 79/101) were identified as PPNG. The annual prevalence of PPNG was 81.6% (31/38) in 2022, 75.0% (27/36) in 2023, and 77.8% (21/27) in 2024. African-type plasmids dominated the PPNG isolates (92.4%, 73/79) during the study period, while only 7 isolates (7.6%, 6/79) contained Asian plasmids (1 in 2022, 2 in 2023, and 3 in 2024). No Toronto/Rio plasmids or other rare plasmid types were detected in this study. Further analysis revealed that only 4 isolates (5.1%, 4/79) among the PPNG carried *bla*_TEM−135_ (2 in 2023, and 2 in 2024), the remaining 75 strains (94.9%, 75/79) carried *bla*_TEM−1_. Moreover, all *bla*_TEM−135_ PPNG isolates contained Asian plasmids.

### Antimicrobial susceptibility of PPNG isolates

The PPNG isolates from the current study could be grouped into three major clusters based on plasmid type and *bla*_TEM_ gene subtype: Asian-type plasmids carrying *bla*_TEM−135_ (*n* = 4), Asian-type plasmids carrying *bla*_TEM−1_ (*n* = 2), and African-type plasmids carrying *bla*_TEM−1_ (*n* = 73). The MIC_50_, MIC_90_, and MIC range of penicillin, ceftriaxone, and cefixime are shown in Table 1. The MICs revealed that 84.2% (85/101), 11.9% (12/101), and 18.8% (19/101) of *Neisseria gonorrhoeae* isolates had resistance to penicillin, ceftriaxone and cefixime, respectively. As expected, PPNG isolates exhibited a markedly higher penicillin resistance rate than non-PPNG isolates. African-type bla_TEM−1_-positive PPNG isolates exhibited higher resistance rates to ceftriaxone (12.3% vs. 9.1%; *p* = 1.000) and cefixime (20.5% vs. 13.6%; *p* = 0.759) compared to non-PPNG strains; the differences were not statistically significant. Because the Asian-type plasmid group contained too few isolates carrying *bla*_TEM−1_ or *bla*_TEM−135_, no statistical comparisons were performed.

**Table 1:** Minimum inhibitory concentrations (MICs) of antimicrobial agents against penicillinase-producing *Neisseria gonorrhoeae* (PPNG) isolates with different plasmid types in Meizhou, 2022–2024

Group	MIC range	MIC_50_	MIC_90_	S	I	*R*
*Asian*-*bla*_TEM−135_ PPNG (*n* = 4)						
Penicillin	≥ 32	≥ 32	≥ 32	0	0	4
Ceftriaxone	0.015–0.5	0.06	0.5	3	-	1
Cefixime	0.03 - ≥1	0.125	1	3	-	1
*Asian*-*bla*_TEM−1_ PPNG (*n* = 2)						
Penicillin	≥ 32	≥ 32	≥ 32	0	0	2
Ceftriaxone	0.008–0.06	-	-	2	0	-
Cefixime	0.03–0.06	-	-	2	0	-
African-*bla*_TEM−1_ PPNG (*n* = 73)						
Penicillin	0.25 - ≥32	≥ 32	≥ 32	0	1	72
Ceftriaxone	0.015-≥1	0.015	0.5	64	-	9
Cefixime	0.015-≥1	0.06	0.25	58	-	15
Non-PPNG (*n* = 22)						
Penicillin	0.125-≥32	0.5	2	12	4	6
Ceftriaxone	0.015−0.5	0.015	0.15	20	-	2
Cefixime	0.015-≥1	0.125	0.5	19	-	3
Total (*n* = 101)						
Penicillin	0.125-≥32	≥ 32	≥ 32	12	4	85
Ceftriaxone	0.015-≥1	0.015	0.5	89	-	12
Cefixime	0.015-≥1	0.125	0.25	82	-	19

MICs are presented in mg/L. Interpretive categories: S, susceptible; I, intermediate; R, resistant. Breakpoints are based on EUCAST guidelines (v 14.0, 2024)

### Characteristics of PPNG isolates resistant to ceftriaxone and/or cefixime

Among the 79 PPNG isolates, 17.7% (14/79) showed resistance to ceftriaxone or cefixime (MIC>0.125 mg/L) and possessed the mosaic or non-mosaic PBP2 patterns. Specifically, 3 isolates (21.4%, 3/14) carried the non-mosaic PBP2 patterns XIII, while 11 isolates (78.6%, 11/14) carried the mosaic PBP2 patterns XXXIV and LX(Table 2). All isolates carried African-type plasmids with *bla*_TEM−1_, and ST1086 was the predominant type in the mosaic PBP2 pattern.

**Table 2:** Characteristics of different p*enA* alleles in penicillinase-producing *Neisseria gonorrhoeae* (PPNG) isolates with resistance to ceftriaxone or cefixime (MIC > 0.125 mg/L)

penA alleles	plasmid types	TEM	PBP2 pattern	NG-MAST
Mosaic (*N* = 11)	African (11)	TEM−1 (11)	XXXIV(8) LX(3)	1086(5)^a^
Non-mosaic (*N* = 3)	African (3)	TEM−1 (3)	XIII (3)	-^b^

^a^ Mosaic group: ST 1086 predominated

^b^ Non-mosaic group: all STs single

### Molecular epidemiology analysis of PPNG

**Fig. 1:**
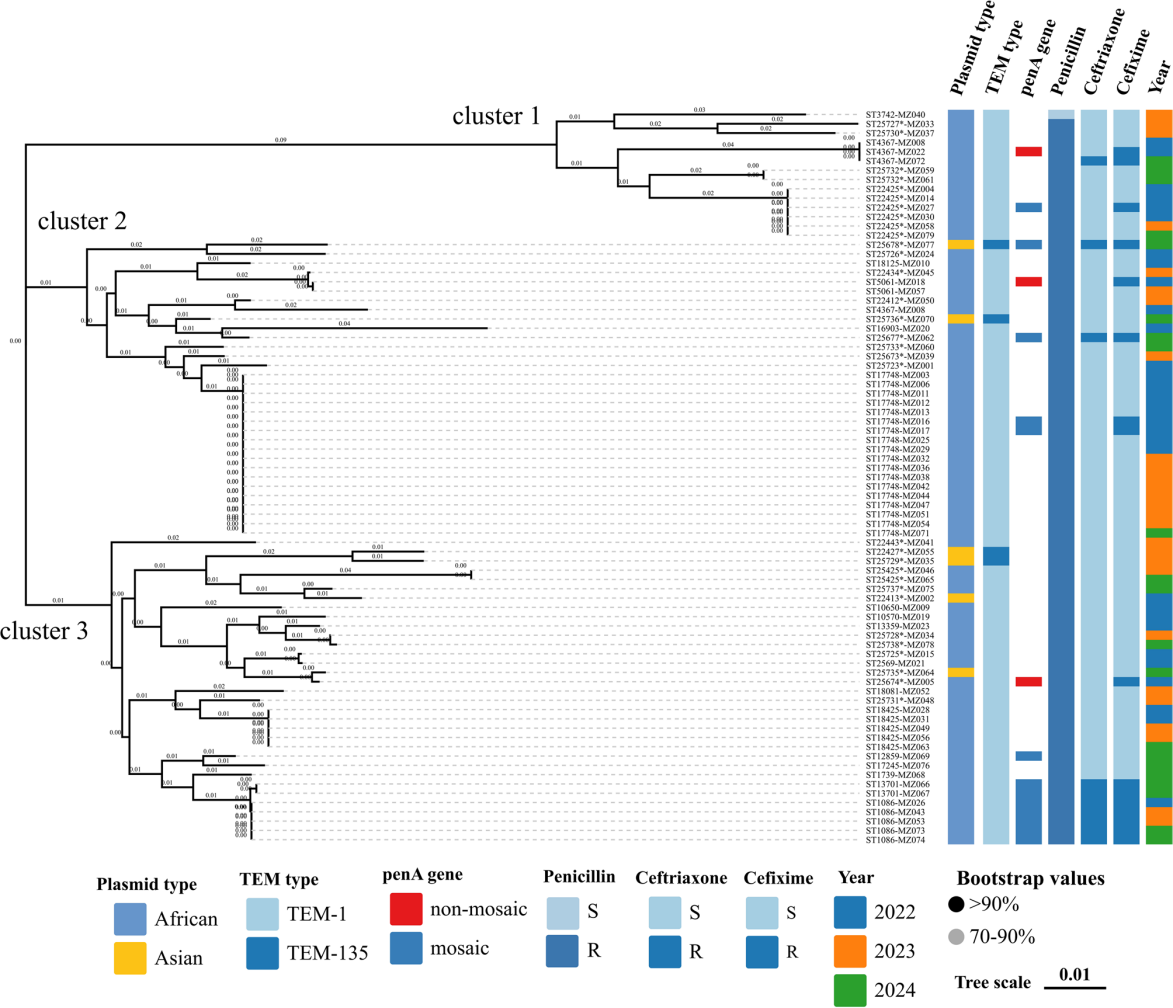
*Neisseria gonorrhoeae* multiantigen sequence typing (NG-MAST) phylogenetic tree of the 79 penicillinase-producing *Neisseria gonorrhoeae* (PPNG) isolates collected in Meizhou between 2022 and 2024. The neighbor-joining tree was constructed in MEGA11 using concatenated *porB* and *tbpB* sequences, with evolutionary distances computed using the Kimura 2-parameter method. * Indicates new NG-MAST sequence types (STs) detected in this study

The 79 PPNG isolates were divided into 43 different NG-MAST STs. Among these isolates, 25 (58.1%, 25/43) novel STs were first identified in the present study, and 9 STs were shared by ≥ 2 isolates. The most prevalent STs were ST17748 (*n* = 18), followed by ST22425 (*n* = 6), ST1086 (*n* = 5), and ST18425 (*n* = 5). Further analysis showed the African-type *bla*_TEM−1_ plasmid was distributed across 37 distinct sequence types, with ST17748 the most common. Notably, all Asian types correspond to new STs. The details of the STs distribution of PPNG isolates with different plasmid types and *bla*_TEM_ genes are shown in the phylogenetic tree in Fig. 1.

### Phylogenetic analyses of PPNG isolates

A phylogenetic tree created by NG-MAST demonstrated that the isolates were primarily clustered into three clusters, indicating heterogeneous epidemiological distribution (Fig. 1). The STs carrying the Asian or TEM-135 plasmids were scattered across the genetic tree in clusters 2 and 3, indicating that these STs represent diverse populations with no close genetic relationship. In cluster 3, ST22427 and ST25729 in the TEM-135 isolates appeared to be closely related based on close genetic distances (determined by NG-MAST) and Asian type (established by plasmid typing). The remaining two isolates harbored the Asian-type *bla*_TEM−135_ plasmid and were assigned to Cluster 2, in which ST17748 was the predominant strain. It is worth noting that all ST1086 strains harbored the African-type *bla*_TEM−1_ plasmid and displayed a mosaic PBP2 pattern, whereas isolate ST25678-MZ077 carried the Asian-type *bla*_TEM−135_ plasmid and shared the same mosaic PBP2 profile. Both genotypes exhibited resistance to penicillin, ceftriaxone, and cefixime.

**Fig. 2:**
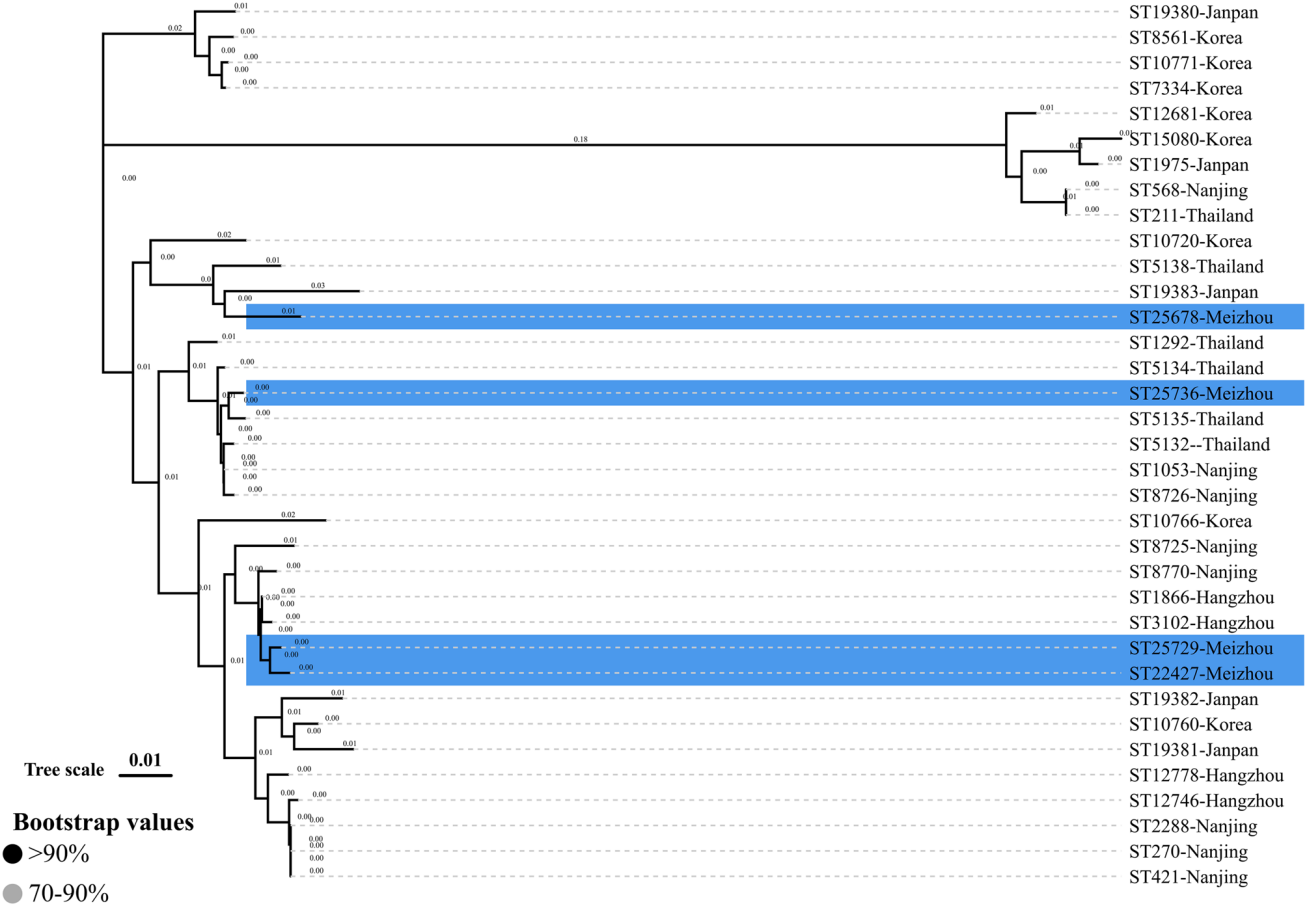
Phylogenetic analysis of *porB* gene sequences from *bla*_TEM−135_ carrying Penicillinase-Producing *Neisseria gonorrhoeae* (PPNG) strains. The tree includes four strains collected in Meizhou between 2022 and 2024 (highlighted in blue) and publicly available *bla*_TEM−135_-positive PPNG strains from Thailand, Japan, South Korea, Hangzhou (China), and Nanjing (China) as controls. The neighbor-joining tree was constructed in MEGA11 based on partial *porB* gene sequences, with evolutionary distances computed using the Kimura 2-parameter method. Each strain is annotated with its sequence type and geographic origin

The phylogenetic analysis of *bla*_TEM−135_-positive isolates based on partial *porB* gene sequences revealed distinct clustering patterns. ST25678 showed close relatedness to ST19383 from Japan, while ST25736 clustered closely with ST5135 from Thailand. Additionally, ST25729 and ST22427 exhibited phylogenetic proximity to ST8770 and ST8725 from Nanjing, as well as ST1866 and ST3102 from Hangzhou. Further details can be found in Fig. 2.

## Discussion

This study revealed that Meizhou’s PPNG prevalence (78.2%, 2022–2024) was significantly higher than the historical level reported for Guangdong Province (47.88%, 2013–2022; *p* < 0.001) [30]. It also surpassed contemporary reports from other Chinese regions, including Nanjing (43.4%, 2014–2018; *p* < 0.001) [31], Hangzhou (35.6%, 2015–2017; *p* < 0.001) [12], as well as those from developed nations such as Japan (1.8%, 2007–2018; *p* < 0.001) [8], South Korea (11.6%, 2005–2015; *p* < 0.001) [13], Italy (18.7%, 2019; *p* < 0.001) [32], England and Wales (4.6%, 2012; *p* < 0.001) [9] and Poland (6.3%, 2010–2014; *p* < 0.001) [33]. Notably, Meizhou’s prevalence closely matched levels observed in high-prevalence settings across Asia and Africa, particularly Thailand (85.5%, 2015–2017; *p* = 0.215) [10], Kenya (72.0%, 2013–2018; *p* = 0.495) [34], and Tanzania (73.7%, 2014–2016; *p* = 0.464) [35]. Because recent studies on PPNG prevalence in other regions are lacking, cross-regional comparisons in this study involve different time frames. However, the PPNG prevalence in Meizhou may reflect either consistently high rates or a gradually increasing trend, which indicates the necessity for continuous monitoring and intervention. Despite the withdrawal of penicillin from gonorrhea treatment guidelines, it continues to hold epidemiological significance as an indicator drug due to the persistent presence of *bla*_TEM_ plasmid-mediated resistance in PPNG strains.

During the study period, two plasmid types were found. The African-type plasmid demonstrated clear predominance over the Asian-type plasmid. In China, the predominant plasmid types among PPNG isolates have shifted significantly. Earlier studies reported Asian-type plasmids as the most prevalent [11, 12, 22, 36], but current data show African-type plasmids now dominate in this region, consistent with recent reports. Specifically, African-type plasmids mainly carry *bla*_TEM−1_, which is consistent with global findings [9, 30]. While Asian-type plasmids may carry *bla*_TEM−1_ or *bla*_TEM−135_, all *bla*_TEM−135_ producing strains in this study exclusively associate with Asian-type plasmids, a pattern supported by several studies [9, 11, 12]. This contrasts with observations in other countries [7], where TEM-135 primarily links to Toronto/Rio plasmids. These differences indicate that the distribution patterns of *bla*_TEM_ gene carrying plasmids exhibit significant geographic variability.

The presence of the *bla*_TEM_ gene, which encodes β-lactamase and mediates penicillin resistance, can account for the significantly higher resistance to penicillin observed in African/*bla*_TEM−1_ PPNG isolates compared to non-PPNG isolates. A previous study showed that the expression levels of the *bla*_TEM_ gene vary significantly [12], leading to differences in the MICs of isolates carrying the *bla*_TEM−1_ and *bla*_TEM−135_ against penicillin, ceftriaxone, and cefixime [8, 11, 13]. Due to the limited number of isolates in this study, it was difficult to ascertain whether there were differences in the expression levels of the *bla*_TEM_ gene across different plasmid types. The observed phenomenon may be attributed to the enhanced thermostability and improved aggregation of TEM-135 β-lactamase, consequently leading to increased intracellular TEM levels and activity [12].

As for PPNG and non-PPNG strains, PPNG shows no difference in susceptibility to ceftriaxone and cefixime, which is consistent with previous reports [7, 30]. In addition to the production of β-lactamase, the causes of penicillin resistance also include mutations in chromosomal genes (mainly the *penA* gene). For strains exhibiting chromosomal resistance to penicillin, the penicillin MIC typically ranges between 2.0 and 4.0 mg/L, while for β-lactamase-producing strains, the MIC values range from 4.0 to 32.0 mg/L [15]. It is important to note that *penA* mutations in *Neisseria gonorrhoeae* lead to the generation of mosaic and non-mosaic PBP2 patterns, which not only increase penicillin MICs but, more significantly, confer resistance to extended-spectrum cephalosporins (ESCs), such as ceftriaxone and cefixime [37]. Several studies have reported PPNG isolates with reduced sensitivity to ESCs. For example, a Japanese study found that 14.0% of PPNG isolates exhibited reduced sensitivity to cefixime, all harboring *bla*_TEM−1_/African or Asian plasmids with varying PBP2 patterns [8]. Similarly, Lee et al. reported that some PPNG isolates with non-mosaic PBP2 types XIII, IV, or XII showed reduced sensitivity to cefixime (MIC ≥ 0.12 mg/L) or ceftriaxone (MIC ≥ 0.12 mg/L) [38]. In this study, PPNG isolates resistant to ceftriaxone and cefixime exhibited PBP2 patterns of Mosaic XXXIV, LX, or non-mosaic XIII, consistent with these findings.

In this study, a total of 45 NG-MAST STs were identified, including 27 novel genotypes, indicating significant genetic diversity of *Neisseria gonorrhoeae* in Meizhou. The predominant genotypes in this region were ST17748, ST22425, ST1086, and ST18425. These findings are consistent with the report by Qin et al., which identified ST17748 as the main circulating strain in Guangdong Province [30]. Although ST2318 [39], which is widely spread nationwide, and ST1407 [40], a common international strain, are prevalent in other regions, neither was detected in this region. Interestingly, ST1086 strains were previously unreported DSC types (MIC ≥ 0.5 mg/L). Similarly, the ST25678-MZ077 isolate carries the Asian-type *bla*TEM-135 plasmid and mosaic PBP2 chromosomal pattern, a relatively uncommon resistance gene combination in this region. It is recommended to strengthen laboratory surveillance and epidemiological monitoring of these strains to evaluate their potential for dissemination.

In the study of *Neisseria gonorrhoeae* isolates carrying the *bla*_TEM−135_ gene, no STs that are prevalent in other Asian regions, such as Nanjing [29], Hangzhou [12], Japan [8], South Korea [13], and Thailand [23], were detected in Meizhou. This suggests that *bla*_TEM−135_-positive *Neisseria gonorrhoeae* strains in different geographic regions may have evolved independently. Moreover, A phylogenetic tree of partial *porB* gene sequences showed that the *bla*_TEM−135_ carrying isolates were related to those from Japan, Thailand, Hangzhou, and Nanjing. Given the high variability of the *porB* genes in NG-MAST typing, we could not determine whether the isolates with specific STs in this region were imported from other cities. But we can speculate that the specific isolates with phylogenetic relationships may share a common evolutionary origin, which could be associated with overlapping sexual networks.

Although the prevalence of TEM-135 remains low among PPNG isolates in Meizhou, with no novel TEM variants detected, the high endemicity of PPNG in this region is a serious concern. While ceftriaxone remains highly effective for uncomplicated genital infections even when some isolates display MICs above the EUCAST resistance breakpoint, its widespread use, especially at sub-optimal doses or for extragenital infections, could incrementally select for higher MICs and favour the emergence of novel TEM variants, including ESBL producers. Continued surveillance and dose- and site-appropriate therapy are therefore essential to preserve ESC efficacy.

This study has several limitations. First, all samples were collected exclusively from symptomatic patients at a single institution (Meizhou People’s Hospital), potentially missing asymptomatic infections and untreated cases in the community. Second, the lack of standardized data on sexual behavior and treatment history limits the analysis of resistance patterns. Third, strain typing was performed with NG-MAST, whose resolution is limited and may underestimate the true diversity of *Neisseria gonorrhoeae* and obscure fine-scale transmission links. Nevertheless, these findings provide important baseline data on the epidemiology of *Neisseria gonorrhoeae* in Meizhou and will support the future regional antimicrobial resistance surveillance.

## Conclusions

This study revealed a high diversity of plasmid profiles among PPNG strains in Meizhou. The detection of multiple novel STs suggests possible strain importation and local genetic recombination. These findings underscore the need for sustained molecular surveillance of PPNG resistance patterns and *bla*_TEM−135_ variants, as well as the prompt reporting of imported or locally emerged lineages to facilitate evidence-based updates to regional treatment guidelines. Such measures will establish an early-warning framework for potential ESBL-producing *Neisseria gonorrhoeae* and help limit onward transmission.

## Data Availability

The nucleotide sequences of NG-MAST targets (*porB* and *tbpB* genes) generated in this study have been deposited in GenBank under accession numbers PX232833–PX232990. The accession numbers for the four *bla* TEM-135-carrying isolates are PX232863, PX232881, PX232893, and PX232900. The novel NG-MAST sequence types identified in this study have been registered in the PubMLST *Neisseria* typing database (https://pubmlst.org/neisseria/) under isolate IDs: 22412-22413, 22425, 22427, 22434, 22443, 25425, 25673-25678, and 25723-25738. All sequence data will be made publicly available upon publication of this article.

## Abbreviations

ESBL: Extended-spectrum β-lactamase
ESCs: Extended-spectrum cephalosporins
EUCAST: European Committee on Antimicrobial Susceptibility Testing
GD-GASP: Guangdong Gonococcal Antimicrobial Surveillance Program
MIC: Minimum inhibitory concentration
NG-MAST: *Neisseria gonorrhoeae* multiantigen sequence typing
PPNG: Penicillinase-Producing *Neisseria gonorrhoeae*
SNP: Single nucleotide polymorphism
ST: Sequence type
STD: Sexually transmitted disease
WHO: World Health Organization
